# Towards legitimacy of the solar geoengineering research enterprise

**DOI:** 10.1098/rsta.2016.0459

**Published:** 2018-04-02

**Authors:** Peter C. Frumhoff, Jennie C. Stephens

**Affiliations:** 1 Union of Concerned Scientists, Cambridge, MA 02138, USA; 2 School of Public Policy and Urban Affairs, Global Resilience Institute, Northeastern University, Boston, MA 02115 USA

**Keywords:** solar geoengineering, climate, Paris Agreement, research co-production, legitimacy

## Abstract

Mounting evidence that even aggressive reductions in net emissions of greenhouse gases will be insufficient to limit global climate risks is increasing calls for atmospheric experiments to better understand the risks and implications of also deploying solar geoengineering technologies to reflect sunlight and rapidly lower surface temperatures. But solar geoengineering research itself poses significant environmental and geopolitical risks. Given limited societal awareness and public dialogue about this climate response option, conducting such experiments without meaningful societal engagement could galvanize opposition to solar geoengineering research from civil society, including the most climate vulnerable communities who are among its intended beneficiaries. Here, we explore whether and how a solar geoengineering research enterprise might be developed in a way that promotes legitimacy as well as scientific credibility and policy relevance. We highlight the distinctive responsibilities of researchers and research funders to ensure that solar geoengineering research proposals are subject to legitimate societal review and scrutiny, recommend steps they can take to strive towards legitimacy and call on them to be explicitly open to multiple potential outcomes, including the societal rejection or considerable alteration of the solar geoengineering research enterprise.

This article is part of the theme issue ‘The Paris Agreement: understanding the physical and social challenges for a warming world of 1.5°C above pre-industrial levels'.

## 1. Introduction

Despite justifiable optimism about the accelerating pace of the energy transition moving away from fossil fuel reliance towards more renewable-based energy systems [1–3], mounting evidence suggests that even very aggressive reductions in greenhouse gas emissions may be insufficient to meet the Paris Agreement's long-term goal of limiting the increase in global average temperatures to well below 2°C above pre-industrial levels [4–6]. Therefore, vexing questions around whether, when and under what conditions society might also need to consider deploying other large-scale ‘climate interventions’ are now coming to the fore. Over the past 10 years, interest and engagement with these issues has been growing [7–10], building on proposals for deliberate interventions in the climate system that have been considered since the 1960s and 1970s [11–14]. Such climate interventions fall broadly into two categories: technologies to remove carbon dioxide from the atmosphere at scale, and ‘albedo modification’ or solar geoengineering technologies to reflect sunlight and lower surface temperatures. Increasingly, scholars across disciplines are wrestling with the risks, uncertainties and implications of the potential deployment of both suites of technologies [7,8,15–17].

Global-scale solar geoengineering through the proposed injection of reflecting aerosols to the stratosphere to rapidly cool the planet is particularly controversial. To date, climate policy-makers have largely avoided considering it. In the Paris Agreement, parties established the aim to stabilize temperatures by achieving ‘a balance between anthropogenic emissions by sources and removals by sinks of greenhouse gases', that is, by bringing net carbon emissions to zero. This framing acknowledges the potential deployment of carbon dioxide removal technologies, but renders solar geoengineering not ‘policy relevant’ towards meeting the Paris Agreement's long-term temperature goals. Thus, the formally adopted outline of the Intergovernmental Panel on Climate Change (IPCC) Special Report on 1.5°C calls for consideration of carbon dioxide removal technologies, but makes no mention of solar geoengineering [18].

Multiple factors, including deeply uncertain geopolitical risks and ramifications, contribute to policy-makers' reluctance to consider solar geoengineering. The risks and benefits of the deployment of any solar geoengineering programme will be unevenly distributed across the world [19,20], and nations are likely to have widely divergent preferences for whether, when, how and towards what climate goals solar geoengineering technologies should be deployed [21,22].

The controversy over solar geoengineering deployment extends to research involving field experiments to better understand both the efficacy and risks of potential deployment. Proposed experiments entail injecting sulfate aerosols or other reflecting particles into the stratosphere as well as various measures to increase the reflectance of marine clouds. Within the research community, views range from firm opposition to field experiments [23] to cautious endorsement of ‘small-scale atmospheric field experiments with controlled emissions’ [15] to calls for an ambitious ‘large-scale international research effort’ [24] investigating through atmospheric experiments detailed plausible operational scenarios for deployment against various researcher-determined measures of risk and effectiveness in achieving desired climate outcomes [24].

Yet, even discussions of small-scale experiments with limited direct environmental risks raise significant ethical and geopolitical concerns about implied intent, for example, towards further, riskier, larger scale research and deployment [15,25]. Currently, no process exists by which proposed atmospheric experiments might gain informed consent from either governments or civil society stakeholders, nor is there clarity on which stakeholders should be engaged in the consideration of research with such global risks and implications.

In a seminal 2003 paper, Cash *et al.* [26] argued that scientific research is most likely to be effective in informing societal responses to challenging issues when research is perceived by relevant stakeholders to be scientifically credible, salient to decision-makers and legitimate. Establishing legitimacy requires the respectful consideration of stakeholders' divergent values and beliefs. Today, solar geoengineering field research lacks both salience (i.e. policy relevance) and legitimacy.

The salience of solar geoengineering research is likely to increase as the IPCC Special Report on 1.5°C and other studies bring to greater policy-maker attention the low prospects of limiting global climate risks through net emissions reductions alone. But the prospects and possible pathways for establishing solar geoengineering field research as legitimate are less clear. Initiating atmospheric field experiments in the absence of a well-designed process to seek legitimacy could draw considerable negative attention and enhance opposition to them. No well-documented solar geoengineering atmospheric experiments have yet been conducted, perhaps for this reason. But some researchers are now actively designing, planning and advocating for solar geoengineering field experiments, drawing attention and backlash and increasing the deliberate haste with which legitimacy for such experiments will need to be carefully sought [27–30].

In this paper, we explore whether and how a solar geoengineering research enterprise might be developed in a way that promotes legitimacy as well as scientific credibility and policy relevance. We first review the arguments for and against advancing solar geoengineering field research, with a focus on proposals to inject reflecting aerosols into the Earth's upper atmosphere. We highlight the responsibilities of researchers and funders of solar geoengineering research to ensure that research proposals are subject to legitimate societal review and scrutiny and recommend steps they can take to strive towards legitimacy of the solar geoengineering research enterprise.

## 2. The case for solar geoengineering field research

Today, a relatively small number of researchers and funders, primarily in industrialized countries, are pressing for accelerated development of solar geoengineering field research to assess the risks and potential benefits of various technologies and deployment scenarios [24,27]. Their case for advancing field research is grounded in the evidence of the serious risks of climate change, growing awareness of the dim prospect that the Paris Agreement's temperature targets will be met through aggressive reductions in net emissions alone and climate model results suggesting how solar geoengineering might supplement aggressive net emissions reductions in limiting climate risks [17].

Keith [24] puts the ethical case for a large-scale international solar geoengineering field research enterprise in environmental justice terms, arguing that the beneficiaries of such a research programme would include ‘the world's most vulnerable people, who lack the resources to move or adapt’ to rising sea levels and increasing extreme weather. Thus, the multiple ‘reasons for reluctance’ which constrain support for solar geoengineering research must be weighed ‘against the evidence that solar geoengineering could avert harm to some of the world's most vulnerable people’.

The scientific case for solar geoengineering field research is grounded in the recognition of the limits of policy-relevant information on efficacy and risks that can be gleaned from observational studies of volcanic plumes and climate model simulations [22]. Advancing knowledge about how aerosol injection into the atmosphere would play out in the field, and improving understanding about impacts and risks, would inform preparations for future scenarios in which solar geoengineering deployment might be considered more prominently than it is today. Modelling studies of projected climate change and the impacts of the sustained deployment of sunlight-reflecting aerosols are valuable, but models alone are insufficient to provide reliable, quantitative information about the relative risks, consequences and benefits of albedo modification to the Earth system as a whole or to the distribution of risks and benefits to specific regions and nations [15].

Small-scale field experiments to release aerosols or their precursors into the atmosphere could reduce climate model uncertainties related to deployment and could arguably be designed to pose low environmental risk [15,27]. Such experiments could contribute to societal understanding of the potential effects, both intended and unintended, of sustained deployment.

Atmospheric experiments could also enhance understanding of how deployment could be detected if carried out unilaterally without international agreement. Unilateral deployment is a plausible scenario. The NRC (2015) assessment explains [15]: ‘As a matter of physical and economic capability a single nation, a large corporation or a group of individuals with sufficient means could potentially deploy albedo modification in the absence of an international consensus or coordination…. Such attempts might begin at small scales…or as an attempt to modify regional climate (e.g. an attempt to restore a failed Indian monsoon or to ameliorate a severe European heat wave)’. It may be feasible to focus research and deployment at addressing such regional-scale climate risks through, for example, tropospheric-level interventions [31].

To effectively assess and detect both anticipated and unanticipated impacts relevant to regional and global-scale deployment, climate response tests of increasing spatial scale, duration and aerosol concentration could be designed [32]. Such research would need to be accompanied by the expansion of a robust global monitoring system [15]. Given that indirect and unanticipated effects could be of greater impact than the direct effects, sophisticated global monitoring of the Earth system responses to field experiments is essential to maximize learning [15,33,34].

## 3. Arguments against solar geoengineering field research

An array of concerns about the risks of advancing solar geoengineering field research have been articulated, and most assume a blurry line, as well as a slippery slope, between research and deployment. Much of the opposition to solar geoengineering field research is aligned with opposition to deployment. Given that investments in research can create momentum towards deployment, and the historical precedence of researchers becoming advocates for deploying the technologies they work on [35,36], there is concern that advancing research will increase the likelihood that solar geoengineering will ultimately be deployed, regardless of what the research reveals about the distribution of societal risks and benefits of deployment.

Opposition ranges from the view that solar geoengineering is inherently undesirable and ungovernable [37,38], to the view that it is a futile effort to control nature [39], to more operational concerns that research would be a waste of resources due to the argued inability of solar geoengineering strategies to actually reduce local- and regional-scale climate risks [37].

Another slippery slope concern about solar geoengineering research relates to the short lifetimes of aerosol particles in the atmosphere, which necessitates a sustained, long-term locked-in effort [36]. If a large-scale solar geoengineering programme were suddenly terminated, the warming offset by the programme would occur rapidly [27]. Given that many climate risks scale with the rate of change, an abruptly halted solar geoengineering programme could bring more severe risks than if the geoengineering programme had never been initiated and the same extent of warming had occurred more gradually [27].

Additional concerns centre on the ‘moral hazard’ of how research on, and even discussion of, solar geoengineering might be a distraction, promoting complacency and further undermining already insufficient national and global political will to mitigate and adapt [37].

Concerns about the challenges of global governance associated with solar geoengineering are particularly relevant to the importance of seeking legitimacy [20]. Any global-scale geoengineering programme can be expected to have regional differences in effectiveness in offsetting climate risks [19,40], with more powerful countries seeking control over both research agendas and deployment plans.

Power struggles over whose preferences are prioritized in a geoengineered world appear likely. It is easy to imagine, for example, the USA seeking outcomes that maintain favourable precipitation patterns for Midwest US farmers even if, in doing so, drought in the African Sahel is worsened. It is reasonable, therefore, to fear that competing interests in controlling the global thermostat will exacerbate both inequality and potential conflict.

Beyond national interests, concerns have also been articulated that a research agenda or deployment strategy could be shaped to prioritize specific corporate or military interests and minimize priorities of climate vulnerable people and communities [41–43].

## 4. Enhancing the legitimacy of solar geoengineering field research

Uncertainties over who will control the global thermostat and how the unequal distribution of impacts might be integrated into governance [20] may exacerbate opposition by civil society and climate vulnerable communities and nations; opposition could occur even with proposals for small-scale geoengineering experiments with seemingly low environmental risk [21,25]. It is also reasonable to anticipate (or fear) that once small-scale research initiatives are underway the results of these experiments will build momentum among researchers and funders to address additional policy-relevant questions that can only be answered through larger scale experiments, experiments with greater risks of intended and unintended consequences that transcend political boundaries [34]. No formal global governance infrastructure currently in place is equipped or prepared to address these challenges, nor is there a coherent set of governance frameworks to guide the potential expansion of research [20].

As the IPCC Special Report on 1.5°C and other analyses draw greater attention to the probability and consequences of global temperatures rising above the Paris Agreement targets, practical interest in learning more about the potential of solar geoengineering is likely to expand, so calls for atmospheric field experiments are likely to continue and intensify.

Within this emerging context of expanded and contested advocacy for solar geoengineering field research, governments and civil society organizations well beyond the geographical reach of small-scale experiments are likely to see themselves as stakeholders in their design and implementation. There is, therefore, high value in establishing a systematic process for gaining meaningful international input and informed consent to seek legitimacy for field experiments. This process is likely to be critically important, even for small-scale field experiments that may be contained and limited in their proposed scope and scale.

At its core, such a process would need to provide a platform for collaborative dialogue over, and assessment of, proposed field experiments in the context of researcher and stakeholder perceptions of multiple, competing risks: the severe, perhaps existential, risks of severe climate change impacts associated with global temperature increases above the Paris Agreement targets; the risk that even substantial investments in net emissions reductions and adaptation may be insufficient to limit temperature increases and protect climate vulnerable nations and communities; and the environmental, social and geopolitical risks of solar geoengineering research and deployment intended to ensure that the Paris temperature targets can be met.

It is challenging to consider how to meaningfully engage a broad range of nations and civil society stakeholders in weighing the complex competing risks and uncertainties in the context of divergent values, priorities and understandings. Today, public awareness of solar geoengineering is very low [44]. Some recent research on public perceptions of and attitudes towards geoengineering reveal that initial perceptions are often negative and sceptical [45–47], and ambiguity and ambivalence are prevalent in public discourse [48]. At this early stage of public discussion, opinions are likely to be fluid and susceptible to change depending on how policy-makers and stakeholders are engaged in understanding and presenting climate risks, the potential of other climate mitigation strategies and the uncertain risks and potential of geoengineering. Recent research on perceptions of geoengineering governance suggests that ‘controllability’ of geoengineering is a central concern [49].

While advocating for the rapid expansion of solar geoengineering field research, Parson *et al.* [27] address concerns over legitimacy as ‘a practical problem of governance and research programme design’, one focused on how research programmes can ‘be designed, funded, and managed to ensure maintenance of legitimate societal control’ over a programme's continuance or expansion.

Their approach could be interpreted as implying that the desired outcome of stakeholder engagement is societal consent and endorsement for an ongoing, expansive solar geoengineering research programme. But if the process of seeking societal input is itself to be legitimate, it must be explicitly open to other outcomes, including the rejection of the solar geoengineering research enterprise or its considerable alteration.

It may be that some of the most climate vulnerable constituencies, who Keith [24] forcefully argues should be viewed as the primary beneficiaries of a responsible solar geoengineering research programme, will come to a different perception of the relative risks and benefits of research and potential deployment from the scientists, policy analysts and funders advocating for atmospheric field experiments. Just as risks of climate change fall most forcefully on the least resilient individuals and communities in the most vulnerable regions of the world, so too will the environmental and geopolitical risks and uncertainties of adding solar geoengineering to the climate mitigation policy toolbox.

Lessons from the history of another contentious climate debate over the role of industrialized-country-led efforts to slow tropical deforestation suggest that prospects for establishing legitimacy of solar geoengineering research will be far greater if developing nations play a leading role in decision-making on geoengineering research design, funding and governance (box 1). We suggest that this should include a process by which research priorities and a set of research standards are co-produced with meaningful input from technical experts, social scientists and civil society organizations from the global south in partnership with their counterparts in high-carbon-emitting nations. The participation of nations fully committed to ambitious emissions reductions and climate adaptation is particularly important so that solar geoengineering research is consistently framed as a potential complement to, rather than a substitute for, other essential efforts to respond to climate change.

Box 1. **The importance of ‘who decides’ in determining the legitimacy of controversial climate mitigation measures.**

The history of a contentious debate over the role of tropical forests in climate mitigation offers insight into the importance of ‘who decides’ in determining the legitimacy of controversial climate mitigation measures. In the late 1990s through early 2000s, a contentious debate centred on whether projects aimed at slowing deforestation in developing countries would be included in the clean development mechanism (CDM) of the Kyoto Protocol. Part of the architecture of the Protocol when it was adopted in 1997, the CDM was designed to allow industrialized countries with binding targets to reduce emissions to meet their commitments in part through clean energy and other emissions reductions projects in developing countries. Left undecided at the time of the Protocol's adoption was the question of whether measures to slow deforestation would be eligible for carbon credits [50].

For several years, proposals to include ‘avoided deforestation’ projects in the CDM were forcefully supported by a coalition of primarily US-based environmental and conservation non-governmental organizations (NGOs), funders and scientists. Advocates of including avoided deforestation pointed to the large contribution of global carbon emissions from tropical deforestation (approx. 20% of the source of annual anthropogenic carbon emissions at the time), the forest and biodiversity conservation co-benefits of slowing deforestation and the technical feasibility of accurately measuring and monitoring reductions in emissions from forests [51,52]. They acknowledged risks and uncertainties, but, focusing on benefits, argued that CDM projects to reduce deforestation would complement, not substitute for, needed energy sector reductions in emissions from the USA and other major industrialized countries. They argued that including this approach would help tropical countries and forest communities meet their own forest conservation and sustainable development goals.

Focusing on risks, forceful opposition to including forestry projects in the CDM came from several prominent international conservation NGOs and forest-rich countries, including Brazil. They viewed the motivations of proponents with suspicion, seeing avoided deforestation projects as a means by which the USA and other industrialized nations might ‘get off the hook’ from having to reduce their own profligate emissions. And they raised fears over sovereignty and control, arguing that forestry projects designed to meet the climate mitigation goals of industrialized nations could pose significant ecological and social harm to forest-rich countries and forest communities that might have little say over project design and implementation [53].

The arguments of opponents were politically persuasive and, in 2001, negotiators dropped efforts to include forestry projects in the CDM [54].

Several years later, a small group of rainforest nations led by Papua New Guinea and Costa Rica stepped forward to embrace and forcefully advocate for climate mitigation measures aimed at slowing tropical deforestation. Under a new policy rubric, championed by developing countries to meet their conservation and development goals, an idea to which many were deeply hostile previously became widely endorsed. Today, support for measures to reduce emissions by protecting and restoring tropical forests is largely uncontroversial and firmly established within the Paris Agreement [55].

The tropical deforestation case reminds us that legitimacy depends in part on the participation of trusted actors. Establishing legitimacy of solar geoengineering research will probably only be achieved once there is an international coalition of collaborators that includes both vulnerable developing nations and major emitting nations unequivocally committed to domestic emissions reductions.

In the absence of established, internationally recognized formal governance regimes for solar geoengineering field research, the responsibilities for ensuring that research proposals are subject to legitimate societal review and scrutiny fall to researchers and research funders.

Hubert *et al.* [56] propose codifying best practices for self-governance into an international ‘code of conduct’ for geoengineering research. Such a code, they argue, should be designed to guide responsible research, inform the development of regulatory processes and promote ‘precaution, risk assessment, public participation and transparency’.

A code of conduct for responsible geoengineering research, recognized and adhered to by researchers and funders of research across nations, would provide an important framework for ensuring that concerns over the legitimacy of solar geoengineering field research are effectively addressed. Towards that end, we suggest that such a code of conduct should call on researchers and funders to meaningfully engage a broad cross-section of civil society stakeholders; be transparent about sources of funding and only accept funding from governments and other entities that are fully committed to deep reductions in greenhouse gas emissions; and accept oversight from an independent advisory committee constituted to review adherence to research and public engagement guidelines.

We briefly elaborate on each of these elements below.

*Meaningfully engage a broad cross-section of civil society stakeholders*. Striving for legitimacy requires researchers and research funders to engage with stakeholders that bring diverse and representative perspectives and priorities in a collaborative process to consider risks and benefits of field research. This should include stakeholders well beyond the geographical scope of the field research who may reasonably fear that initial, bounded, relatively low-risk research is a stepping stone to expanded and riskier research and potential deployment with larger scale (global) implications.

**Figure 1.**
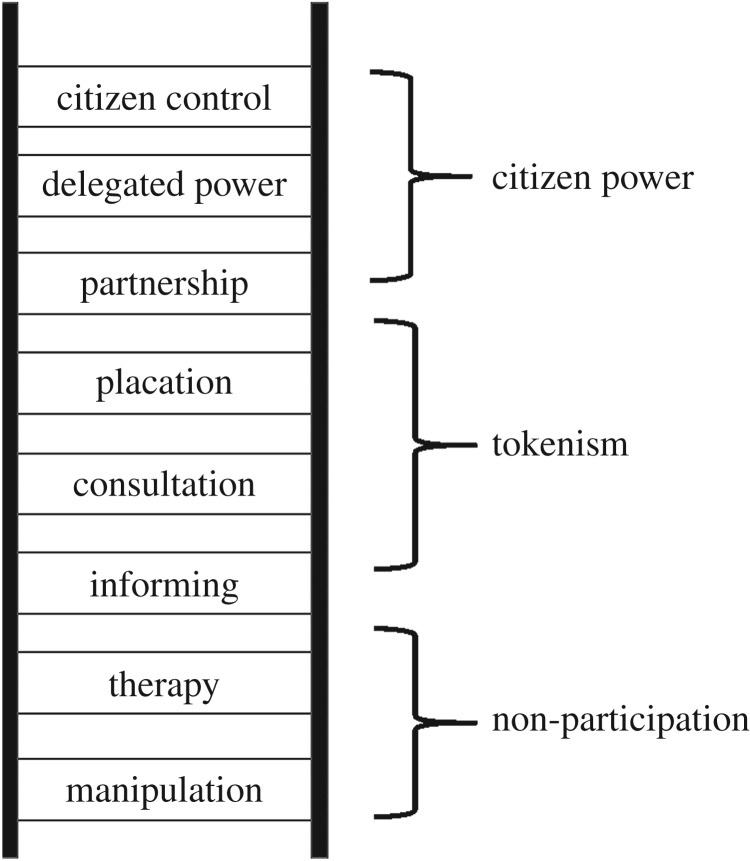
Arnstein's ladder of citizen participation. Redrawn from Arnstein [57], by permission of The American Planning Association (www.planning.org) and Taylor & Francis Ltd (http://www.informaworld.com).

In a classic paper, Arnstein [57] described variation along the ‘ladder of citizen participation’ (figure 1), highlighting that the specific characteristics of stakeholder engagement processes can vary greatly and result in vastly different outcomes in terms of legitimacy. She provocatively distinguished between going through the empty ritual of participation when less powerful stakeholders are either not actively participating or offered token participation through information, consultation or placation, and the very different situation when power is equitably distributed within the process. When considering a stakeholder engagement process surrounding solar geoengineering field research, researchers, research funders and those advocating for field research may have considerably more power than civil society stakeholders or representatives of climate vulnerable communities.

Participation can be an empty and frustrating process for those with little power. An engagement process that is not near the top of Arnstein's ladder may therefore reduce, rather than strengthen, legitimacy.

Engagement processes should provide opportunities for meaningful civil society input into research design and implementation, including, potentially, rejection or significant alteration of the proposed research. A rich literature has developed on the value of co-production of research agendas and co-design of research approaches between scientists and stakeholders [58]. This co-production model can be adapted and incorporated into the stakeholder engagement process focused on collectively reflecting on the risks and benefits of atmospheric field research.

*Be transparent about sources of funding and only accept funding from governments and other entities that are fully committed to deep reductions in greenhouse gas emissions.* Efforts to establish solar geoengineering field research as legitimate would be undermined should research funding not be transparent or sourced from governments, industries or other entities not fully committed to deep emissions reductions as the priority means of achieving the Paris Agreement's long-term climate objectives. To do otherwise would exacerbate ‘moral hazard’ concerns that solar geoengineering will be deployed as a substitute for swift and aggressive reductions in net emissions [33].

*Accept oversight from an independent advisory committee constituted to review adherence to research and public engagement guidelines.* External evaluation and oversight on how well the code of conduct is being applied will be important for strengthening legitimacy. An external advisory committee could also be charged with ensuring that lessons from initial experiments inform the development of any further solar geoengineering field research initiatives.

Ideally, a standing independent advisory committee should be developed to provide oversight to multiple independent solar geoengineering research initiatives, allowing deliberative learning and information sharing and ongoing capacity. Such a committee might be formed, for example, through an already established ‘boundary organization’, such as the Carnegie Climate Geoengineering Governance Initiative (C2G2), launched in February 2017 to ‘encourage society-wide discussion about the risks, potential benefits, ethical and governance challenges raised by climate geoengineering’ [59], or the Solar Radiation Management Governance Initiative (SRMGI), a partnership launched by the Royal Society, The World Academy of Sciences (TWAS) and the Environmental Defense Fund (EDF), established to bring developing countries' voices into discussions on how solar geoengineering research might be governed [60].

## 5. Conclusion

Public dialogue around solar geoengineering research and potential deployment is at a very early stage with considerable uncertainty over how different stakeholders will come to view its potential and its risks [49]. It is by no means certain that the urgency with which the need for atmospheric field research is felt within some quarters of the climate science community will be shared more broadly among expert communities, by political leaders or by the various publics whose vulnerabilities to climate change impacts justify the case for expansive research. Now is the time, therefore, in advance of any field research, for the solar geoengineering research community to strive for societal legitimacy.

In his critique of ‘opening up’ the governance of science and technology, Stirling [61] highlighted three different intentions in advocating for participatory processes: (i) the normative intention that it is the right thing to do, (ii) the operational intention that the participatory process will enable a goal to be achieved, and (iii) the substantive intention that recognizes the value of integrating diverse perspectives into the outcome. We draw upon each of these intentions in justifying our proposed suggestions for strengthening legitimacy through a systematic engagement process. Normatively, engaging a broader constituency in developing solar geoengineering research norms and guidelines and striving for legitimacy is the right thing to do. Operationally, proceeding with atmospheric field experiments at any scale without first establishing a legitimate collaborative process of research co-design and co-production with stakeholders could jeopardize responsible societal consideration of solar geoengineering as an approach to reduce the risks of catastrophic climate change. And, substantively, integrating diverse perspectives and expertise into the consideration of whether and how solar geoengineering research should proceed will surely strengthen the design, quality and capacity to equitably inform diverse societal goals of any solar geoengineering field research programme that might—or might not—legitimately ensue.

We conclude that solar geoengineering field research should not take place unless and until greater societal legitimacy has been established. We strongly encourage researchers and funders eager to move forward with solar geoengineering field research to support, participate in and await the outcomes of these processes and be explicitly open to multiple potential outcomes, including the societal rejection of field research or its considerable alteration.

## Data accessibility

This article has no additional data.
